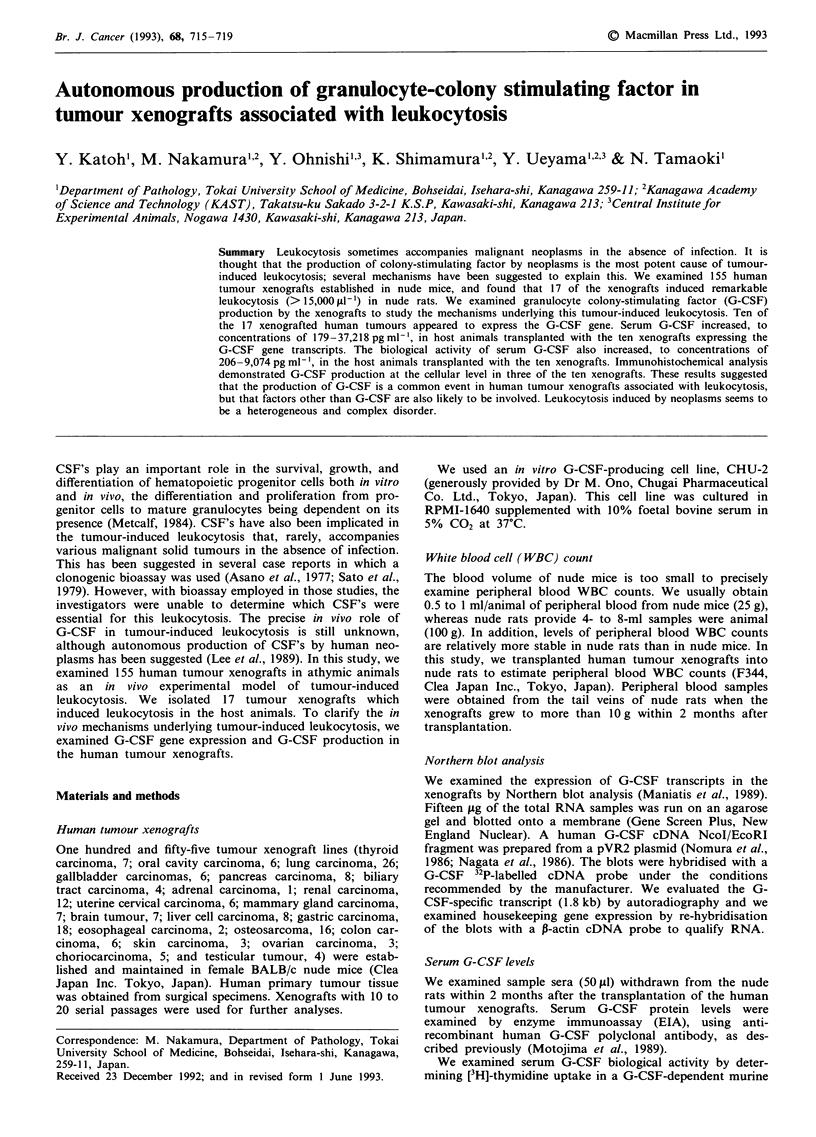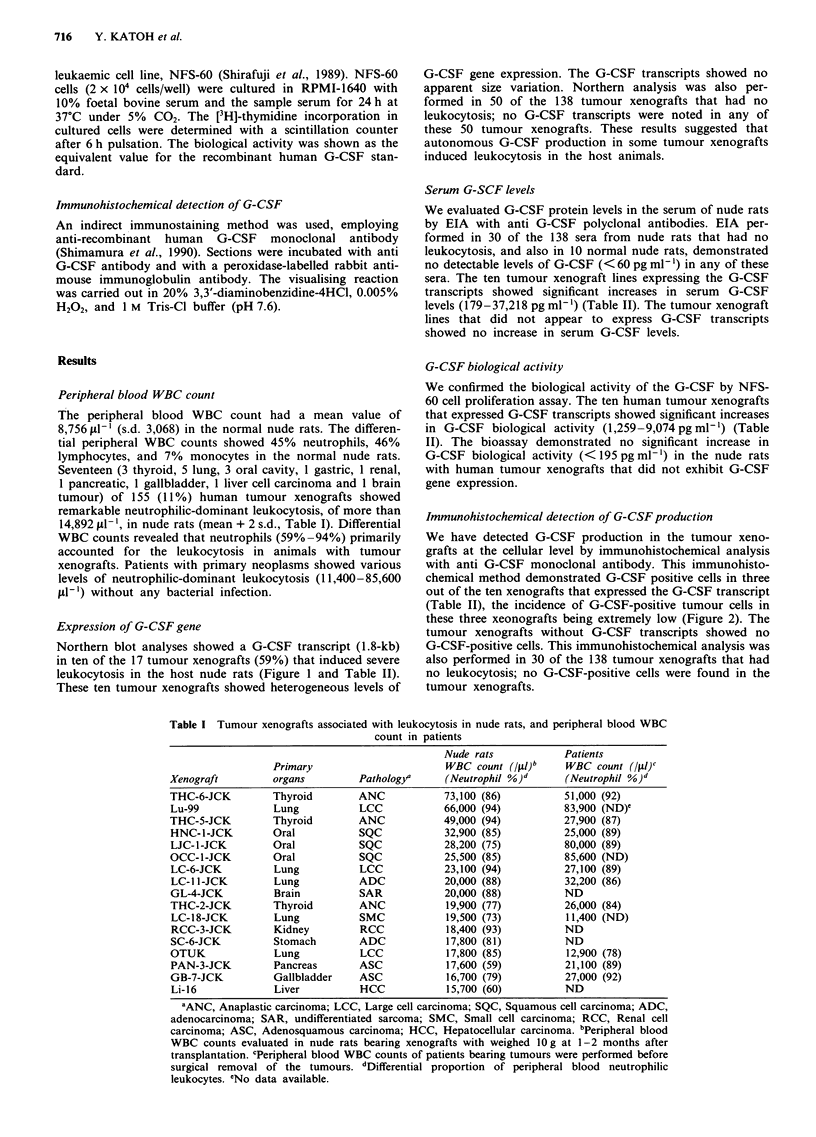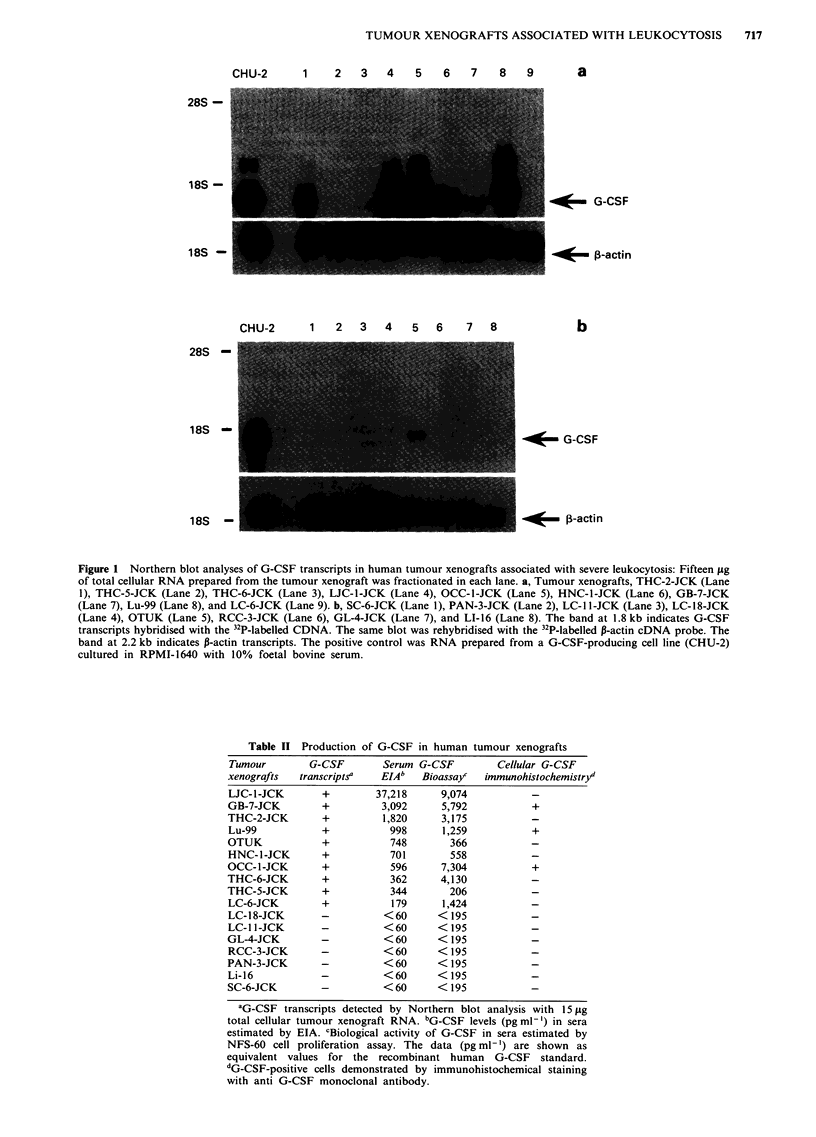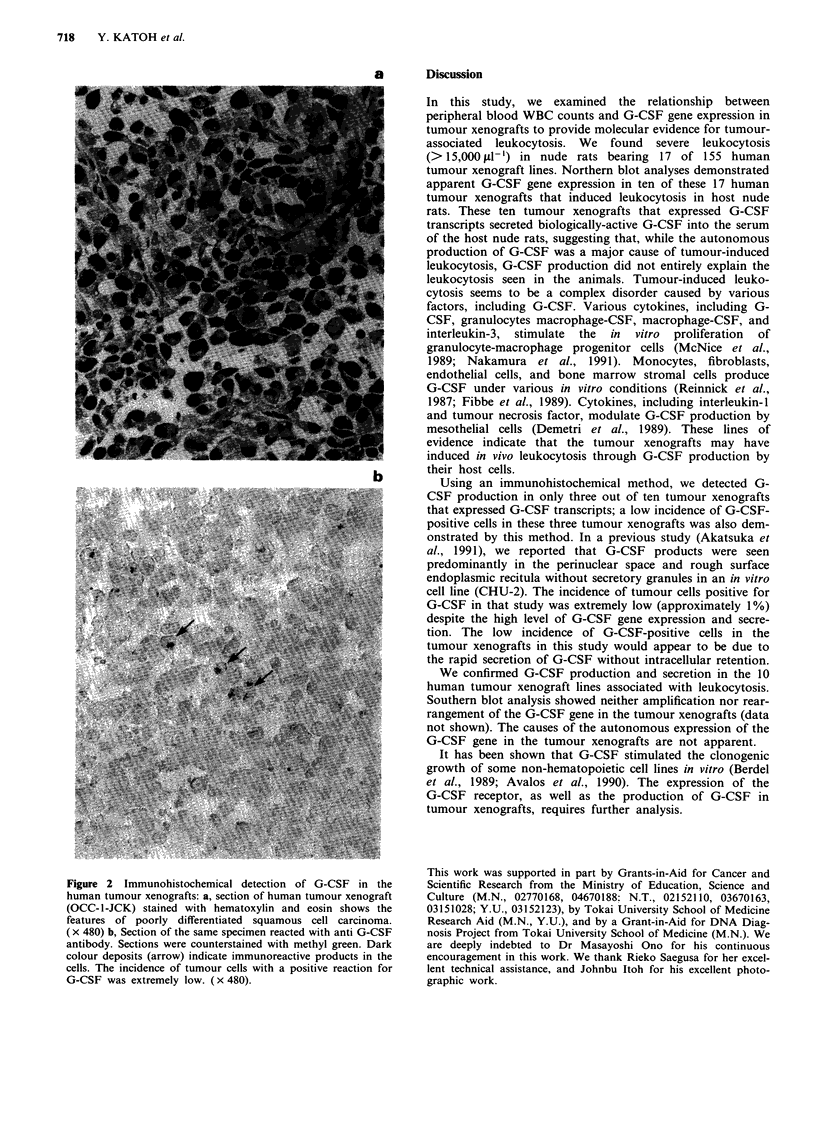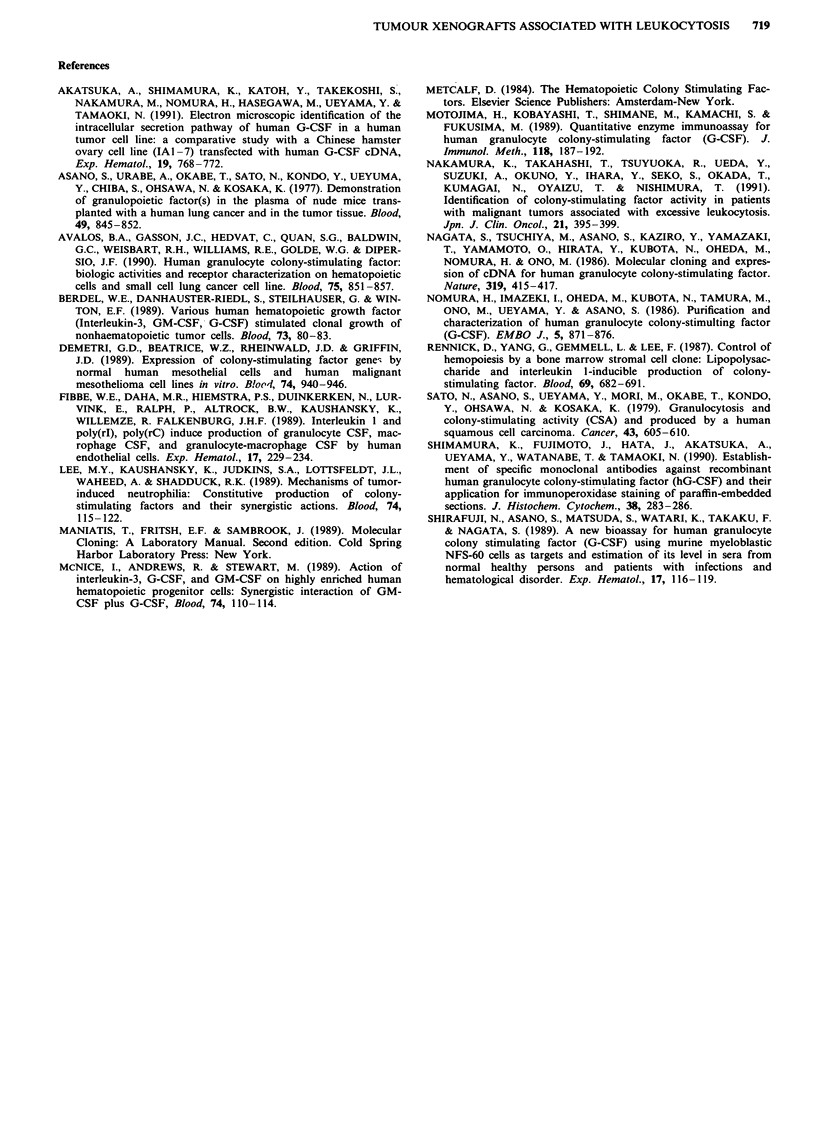# Autonomous production of granulocyte-colony stimulating factor in tumour xenografts associated with leukocytosis.

**DOI:** 10.1038/bjc.1993.416

**Published:** 1993-10

**Authors:** Y. Katoh, M. Nakamura, Y. Ohnishi, K. Shimamura, Y. Ueyama, N. Tamaoki

**Affiliations:** Department of Pathology, Tokai University School of Medicine, Kanagawa, Japan.

## Abstract

**Images:**


Br. J. Cancer (1993), 68, 715-719 © Macmillan Press Ltd., 1993

### Autonomous production of granulocyte-colony stimulating factor in tumour xenografts associated with leukocytosis

Y. Katoh[1], M. Nakamura[1,2], Y. Ohnishi[1,3], K. Shimamura[1,2], Y. Ueyama[1,2,3] & N. Tamaoki[1]

[1]*Department of Pathology, Tokai University School of Medicine, Bohseidai, Isehara-shi, Kanagawa 259-11;* [2]*Kanagawa Academy of Science and Technology (KAST), Takatsu-ku Sakado 3-2-1 K.S.P, Kawasaki-shi, Kanagawa 213;* [3]*Central Institute for Experimental Animals, Nogawa 1430, Kawasaki-shi, Kanagawa 213, Japan.*

**Summary** Leukocytosis sometimes accompanies malignant neoplasms in the absence of infection. It is thought that the production of colony-stimulating factor by neoplasms is the most potent cause of tumour-induced leukocytosis; several mechanisms have been suggested to explain this. We examined 155 human tumour xenografts established in nude mice, and found that 17 of the xenografts induced remarkable leukocytosis ($>15{,}000\,\mu l^{-1}$) in nude rats. We examined granulocyte colony-stimulating factor (G-CSF) production by the xenografts to study the mechanisms underlying this tumour-induced leukocytosis. Ten of the 17 xenografted human tumours appeared to express the G-CSF gene. Serum G-CSF increased, to concentrations of 179–37,218 $pg\,ml^{-1}$, in host animals transplanted with the ten xenografts expressing the G-CSF gene transcripts. The biological activity of serum G-CSF also increased, to concentrations of 206–9,074 $pg\,ml^{-1}$, in the host animals transplanted with the ten xenografts. Immunohistochemical analysis demonstrated G-CSF production at the cellular level in three of the ten xenografts. These results suggested that the production of G-CSF is a common event in human tumour xenografts associated with leukocytosis, but that factors other than G-CSF are also likely to be involved. Leukocytosis induced by neoplasms seems to be a heterogeneous and complex disorder.

CSF's play an important role in the survival, growth, and differentiation of hematopoietic progenitor cells both *in vitro* and *in vivo*, the differentiation and proliferation from progenitor cells to mature granulocytes being dependent on its presence (Metcalf, 1984). CSF's have also been implicated in the tumour-induced leukocytosis that, rarely, accompanies various malignant solid tumours in the absence of infection. This has been suggested in several case reports in which a clonogenic bioassay was used (Asano *et al.*, 1977; Sato *et al.*, 1979). However, with bioassay employed in those studies, the investigators were unable to determine which CSF's were essential for this leukocytosis. The precise *in vivo* role of G-CSF in tumour-induced leukocytosis is still unknown, although autonomous production of CSF's by human neoplasms has been suggested (Lee *et al.*, 1989). In this study, we examined 155 human tumour xenografts in athymic animals as an *in vivo* experimental model of tumour-induced leukocytosis. We isolated 17 tumour xenografts which induced leukocytosis in the host animals. To clarify the *in vivo* mechanisms underlying tumour-induced leukocytosis, we examined G-CSF gene expression and G-CSF production in the human tumour xenografts.

### Materials and methods

### *Human tumour xenografts*

One hundred and fifty-five tumour xenograft lines (thyroid carcinoma, 7; oral cavity carcinoma, 6; lung carcinoma, 26; gallbladder carcinomas, 6; pancreas carcinoma, 8; biliary tract carcinoma, 4; adrenal carcinoma, 1; renal carcinoma, 12; uterine cervical carcinoma, 6; mammary gland carcinoma, 7; brain tumour, 7; liver cell carcinoma, 8; gastric carcinoma, 18; eosophageal carcinoma, 2; osteosarcoma, 16; colon carcinoma, 6; skin carcinoma, 3; ovarian carcinoma, 3; choriocarcinoma, 5; and testicular tumour, 4) were established and maintained in female BALB/c nude mice (Clea Japan Inc. Tokyo, Japan). Human primary tumour tissue was obtained from surgical specimens. Xenografts with 10 to 20 serial passages were used for further analyses.

Correspondence: M. Nakamura, Department of Pathology, Tokai University School of Medicine, Bohseidai, Isehara-shi, Kanagawa, 259-11, Japan.
Received 23 December 1992; and in revised form 1 June 1993.

We used an *in vitro* G-CSF-producing cell line, CHU-2 (generously provided by Dr M. Ono, Chugai Pharmaceutical Co. Ltd., Tokyo, Japan). This cell line was cultured in RPMI-1640 supplemented with 10% foetal bovine serum in 5% $CO_2$ at 37°C.

### *White blood cell (WBC) count*

The blood volume of nude mice is too small to precisely examine peripheral blood WBC counts. We usually obtain 0.5 to 1 ml/animal of peripheral blood from nude mice (25 g), whereas nude rats provide 4- to 8-ml samples were animal (100 g). In addition, levels of peripheral blood WBC counts are relatively more stable in nude rats than in nude mice. In this study, we transplanted human tumour xenografts into nude rats to estimate peripheral blood WBC counts (F344, Clea Japan Inc., Tokyo, Japan). Peripheral blood samples were obtained from the tail veins of nude rats when the xenografts grew to more than 10 g within 2 months after transplantation.

### *Northern blot analysis*

We examined the expression of G-CSF transcripts in the xenografts by Northern blot analysis (Maniatis *et al.*, 1989). Fifteen $\mu g$ of the total RNA samples was run on an agarose gel and blotted onto a membrane (Gene Screen Plus, New England Nuclear). A human G-CSF cDNA NcoI/EcoRI fragment was prepared from a pVR2 plasmid (Nomura *et al.*, 1986; Nagata *et al.*, 1986). The blots were hybridised with a G-CSF $^{32}P$-labelled cDNA probe under the conditions recommended by the manufacturer. We evaluated the G-CSF-specific transcript (1.8 kb) by autoradiography and we examined housekeeping gene expression by re-hybridisation of the blots with a $\beta$-actin cDNA probe to qualify RNA.

### *Serum G-CSF levels*

We examined sample sera (50 $\mu l$) withdrawn from the nude rats within 2 months after the transplantation of the human tumour xenografts. Serum G-CSF protein levels were examined by enzyme immunoassay (EIA), using anti-recombinant human G-CSF polyclonal antibody, as described previously (Motojima *et al.*, 1989).

We examined serum G-CSF biological activity by determining [$^3H$]-thymidine uptake in a G-CSF-dependent murine leukaemic cell line, NFS-60 (Shirafuji et al., 1989). NFS-60 cells ($2 \times 10^4$ cells/well) were cultured in RPMI-1640 with 10% foetal bovine serum and the sample serum for 24 h at 37°C under 5% $CO_2$. The [$^3$H]-thymidine incorporation in cultured cells were determined with a scintillation counter after 6 h pulsation. The biological activity was shown as the equivalent value for the recombinant human G-CSF standard.

### *Immunohistochemical detection of G-CSF*

An indirect immunostaining method was used, employing anti-recombinant human G-CSF monoclonal antibody (Shimamura et al., 1990). Sections were incubated with anti G-CSF antibody and with a peroxidase-labelled rabbit anti-mouse immunoglobulin antibody. The visualising reaction was carried out in 20% 3,3'-diaminobenzidine-4HCl, 0.005% $H_2O_2$, and 1 M Tris-Cl buffer (pH 7.6).

### Results

### *Peripheral blood WBC count*

The peripheral blood WBC count had a mean value of 8,756 $\mu l^{-1}$ (s.d. 3,068) in the normal nude rats. The differential peripheral WBC counts showed 45% neutrophils, 46% lymphocytes, and 7% monocytes in the normal nude rats. Seventeen (3 thyroid, 5 lung, 3 oral cavity, 1 gastric, 1 renal, 1 pancreatic, 1 gallbladder, 1 liver cell carcinoma and 1 brain tumour) of 155 (11%) human tumour xenografts showed remarkable neutrophilic-dominant leukocytosis, of more than 14,892 $\mu l^{-1}$, in nude rats (mean + 2 s.d., Table I). Differential WBC counts revealed that neutrophils (59%–94%) primarily accounted for the leukocytosis in animals with tumour xenografts. Patients with primary neoplasms showed various levels of neutrophilic-dominant leukocytosis (11,400–85,600 $\mu l^{-1}$) without any bacterial infection.

### *Expression of G-CSF gene*

Northern blot analyses showed a G-CSF transcript (1.8-kb) in ten of the 17 tumour xenografts (59%) that induced severe leukocytosis in the host nude rats (Figure 1 and Table II). These ten tumour xenografts showed heterogeneous levels of G-CSF gene expression. The G-CSF transcripts showed no apparent size variation. Northern analysis was also performed in 50 of the 138 tumour xenografts that had no leukocytosis; no G-CSF transcripts were noted in any of these 50 tumour xenografts. These results suggested that autonomous G-CSF production in some tumour xenografts induced leukocytosis in the host animals.

### *Serum G-SCF levels*

We evaluated G-CSF protein levels in the serum of nude rats by EIA with anti G-CSF polyclonal antibodies. EIA performed in 30 of the 138 sera from nude rats that had no leukocytosis, and also in 10 normal nude rats, demonstrated no detectable levels of G-CSF ($< 60$ pg $ml^{-1}$) in any of these sera. The ten tumour xenograft lines expressing the G-CSF transcripts showed significant increases in serum G-CSF levels (179–37,218 pg $ml^{-1}$) (Table II). The tumour xenograft lines that did not appear to express G-CSF transcripts showed no increase in serum G-CSF levels.

### *G-CSF biological activity*

We confirmed the biological activity of the G-CSF by NFS-60 cell proliferation assay. The ten human tumour xenografts that expressed G-CSF transcripts showed significant increases in G-CSF biological activity (1,259–9,074 pg $ml^{-1}$) (Table II). The bioassay demonstrated no significant increase in G-CSF biological activity ($< 195$ pg $ml^{-1}$) in the nude rats with human tumour xenografts that did not exhibit G-CSF gene expression.

### *Immunohistochemical detection of G-CSF production*

We have detected G-CSF production in the tumour xenografts at the cellular level by immunohistochemical analysis with anti G-CSF monoclonal antibody. This immunohistochemical method demonstrated G-CSF positive cells in three out of the ten xenografts that expressed the G-CSF transcript (Table II), the incidence of G-CSF-positive tumour cells in these three xeonografts being extremely low (Figure 2). The tumour xenografts without G-CSF transcripts showed no G-CSF-positive cells. This immunohistochemical analysis was also performed in 30 of the 138 tumour xenografts that had no leukocytosis; no G-CSF-positive cells were found in the tumour xenografts.

**Table I** Tumour xenografts associated with leukocytosis in nude rats, and peripheral blood WBC count in patients

| *Xenograft* | *Primary organs* | *Pathology*[a] | *Nude rats WBC count (/μl)*[b] *(Neutrophil %)*[d] | *Patients WBC count (/μl)*[c] *(Neutrophil %)*[d] |
|---|---|---|---|---|
| THC-6-JCK | Thyroid | ANC | 73,100 (86) | 51,000 (92) |
| Lu-99 | Lung | LCC | 66,000 (94) | 83,900 (ND)[e] |
| THC-5-JCK | Thyroid | ANC | 49,000 (94) | 27,900 (87) |
| HNC-1-JCK | Oral | SQC | 32,900 (85) | 25,000 (89) |
| LJC-1-JCK | Oral | SQC | 28,200 (75) | 80,000 (89) |
| OCC-1-JCK | Oral | SQC | 25,500 (85) | 85,600 (ND) |
| LC-6-JCK | Lung | LCC | 23,100 (94) | 27,100 (89) |
| LC-11-JCK | Lung | ADC | 20,000 (88) | 32,200 (86) |
| GL-4-JCK | Brain | SAR | 20,000 (88) | ND |
| THC-2-JCK | Thyroid | ANC | 19,900 (77) | 26,000 (84) |
| LC-18-JCK | Lung | SMC | 19,500 (73) | 11,400 (ND) |
| RCC-3-JCK | Kidney | RCC | 18,400 (93) | ND |
| SC-6-JCK | Stomach | ADC | 17,800 (81) | ND |
| OTUK | Lung | LCC | 17,800 (85) | 12,900 (78) |
| PAN-3-JCK | Pancreas | ASC | 17,600 (59) | 21,100 (89) |
| GB-7-JCK | Gallbladder | ASC | 16,700 (79) | 27,000 (92) |
| Li-16 | Liver | HCC | 15,700 (60) | ND |

[a]ANC, Anaplastic carcinoma; LCC, Large cell carcinoma; SQC, Squamous cell carcinoma; ADC, adenocarcinoma; SAR, undifferentiated sarcoma; SMC, Small cell carcinoma; RCC, Renal cell carcinoma; ASC, Adenosquamous carcinoma; HCC, Hepatocellular carcinoma. [b]Peripheral blood WBC counts evaluated in nude rats bearing xenografts with weighed 10 g at 1–2 months after transplantation. [c]Peripheral blood WBC counts of patients bearing tumours were performed before surgical removal of the tumours. [d]Differential proportion of peripheral blood neutrophilic leukocytes. [e]No data available.

**Figure 1** Northern blot analyses of G-CSF transcripts in human tumour xenografts associated with severe leukocytosis: Fifteen μg of total cellular RNA prepared from the tumour xenograft was fractionated in each lane. **a**, Tumour xenografts, THC-2-JCK (Lane 1), THC-5-JCK (Lane 2), THC-6-JCK (Lane 3), LJC-1-JCK (Lane 4), OCC-1-JCK (Lane 5), HNC-1-JCK (Lane 6), GB-7-JCK (Lane 7), Lu-99 (Lane 8), and LC-6-JCK (Lane 9). **b**, SC-6-JCK (Lane 1), PAN-3-JCK (Lane 2), LC-11-JCK (Lane 3), LC-18-JCK (Lane 4), OTUK (Lane 5), RCC-3-JCK (Lane 6), GL-4-JCK (Lane 7), and LI-16 (Lane 8). The band at 1.8 kb indicates G-CSF transcripts hybridised with the $^{32}$P-labelled CDNA. The same blot was rehybridised with the $^{32}$P-labelled β-actin cDNA probe. The band at 2.2 kb indicates β-actin transcripts. The positive control was RNA prepared from a G-CSF-producing cell line (CHU-2) cultured in RPMI-1640 with 10% foetal bovine serum.

**Table II** Production of G-CSF in human tumour xenografts

| *Tumour xenografts* | *G-CSF transcripts*[a] | *Serum G-CSF EIA*[b] | *Serum G-CSF Bioassay*[c] | *Cellular G-CSF immunohistochemistry*[d] |
|---|---|---|---|---|
| LJC-1-JCK | + | 37,218 | 9,074 | – |
| GB-7-JCK | + | 3,092 | 5,792 | + |
| THC-2-JCK | + | 1,820 | 3,175 | – |
| Lu-99 | + | 998 | 1,259 | + |
| OTUK | + | 748 | 366 | – |
| HNC-1-JCK | + | 701 | 558 | – |
| OCC-1-JCK | + | 596 | 7,304 | + |
| THC-6-JCK | + | 362 | 4,130 | – |
| THC-5-JCK | + | 344 | 206 | – |
| LC-6-JCK | + | 179 | 1,424 | – |
| LC-18-JCK | – | <60 | <195 | – |
| LC-11-JCK | – | <60 | <195 | – |
| GL-4-JCK | – | <60 | <195 | – |
| RCC-3-JCK | – | <60 | <195 | – |
| PAN-3-JCK | – | <60 | <195 | – |
| Li-16 | – | <60 | <195 | – |
| SC-6-JCK | – | <60 | <195 | – |

[a]G-CSF transcripts detected by Northern blot analysis with 15 μg total cellular tumour xenograft RNA. [b]G-CSF levels (pg ml$^{-1}$) in sera estimated by EIA. [c]Biological activity of G-CSF in sera estimated by NFS-60 cell proliferation assay. The data (pg ml$^{-1}$) are shown as equivalent values for the recombinant human G-CSF standard. [d]G-CSF-positive cells demonstrated by immunohistochemical staining with anti G-CSF monoclonal antibody.

**Figure 2** Immunohistochemical detection of G-CSF in the human tumour xenografts: **a**, section of human tumour xenograft (OCC-1-JCK) stained with hematoxylin and eosin shows the features of poorly differentiated squamous cell carcinoma. (× 480) **b**, Section of the same specimen reacted with anti G-CSF antibody. Sections were counterstained with methyl green. Dark colour deposits (arrow) indicate immunoreactive products in the cells. The incidence of tumour cells with a positive reaction for G-CSF was extremely low. (× 480).

### Discussion

In this study, we examined the relationship between peripheral blood WBC counts and G-CSF gene expression in tumour xenografts to provide molecular evidence for tumour-associated leukocytosis. We found severe leukocytosis ($>15,000\,\mu l^{-1}$) in nude rats bearing 17 of 155 human tumour xenograft lines. Northern blot analyses demonstrated apparent G-CSF gene expression in ten of these 17 human tumour xenografts that induced leukocytosis in host nude rats. These ten tumour xenografts that expressed G-CSF transcripts secreted biologically-active G-CSF into the serum of the host nude rats, suggesting that, while the autonomous production of G-CSF was a major cause of tumour-induced leukocytosis, G-CSF production did not entirely explain the leukocytosis seen in the animals. Tumour-induced leukocytosis seems to be a complex disorder caused by various factors, including G-CSF. Various cytokines, including G-CSF, granulocytes macrophage-CSF, macrophage-CSF, and interleukin-3, stimulate the *in vitro* proliferation of granulocyte-macrophage progenitor cells (McNice *et al.*, 1989; Nakamura *et al.*, 1991). Monocytes, fibroblasts, endothelial cells, and bone marrow stromal cells produce G-CSF under various *in vitro* conditions (Reinnick *et al.*, 1987; Fibbe *et al.*, 1989). Cytokines, including interleukin-1 and tumour necrosis factor, modulate G-CSF production by mesothelial cells (Demetri *et al.*, 1989). These lines of evidence indicate that the tumour xenografts may have induced *in vivo* leukocytosis through G-CSF production by their host cells.

Using an immunohistochemical method, we detected G-CSF production in only three out of ten tumour xenografts that expressed G-CSF transcripts; a low incidence of G-CSF-positive cells in these three tumour xenografts was also demonstrated by this method. In a previous study (Akatsuka *et al.*, 1991), we reported that G-CSF products were seen predominantly in the perinuclear space and rough surface endoplasmic recitula without secretory granules in an *in vitro* cell line (CHU-2). The incidence of tumour cells positive for G-CSF in that study was extremely low (approximately 1%) despite the high level of G-CSF gene expression and secretion. The low incidence of G-CSF-positive cells in the tumour xenografts in this study would appear to be due to the rapid secretion of G-CSF without intracellular retention.

We confirmed G-CSF production and secretion in the 10 human tumour xenograft lines associated with leukocytosis. Southern blot analysis showed neither amplification nor rearrangement of the G-CSF gene in the tumour xenografts (data not shown). The causes of the autonomous expression of the G-CSF gene in the tumour xenografts are not apparent.

It has been shown that G-CSF stimulated the clonogenic growth of some non-hematopoietic cell lines *in vitro* (Berdel *et al.*, 1989; Avalos *et al.*, 1990). The expression of the G-CSF receptor, as well as the production of G-CSF in tumour xenografts, requires further analysis.

This work was supported in part by Grants-in-Aid for Cancer and Scientific Research from the Ministry of Education, Science and Culture (M.N., 02770168, 04670188; N.T., 02152110, 03670163, 03151028; Y.U., 03152123), by Tokai University School of Medicine Research Aid (M.N., Y.U.), and by a Grant-in-Aid for DNA Diagnosis Project from Tokai University School of Medicine (M.N.). We are deeply indebted to Dr Masayoshi Ono for his continuous encouragement in this work. We thank Rieko Saegusa for her excellent technical assistance, and Johnbu Itoh for his excellent photographic work.

**References**

AKATSUKA, A., SHIMAMURA, K., KATOH, Y., TAKEKOSHI, S., NAKAMURA, M., NOMURA, H., HASEGAWA, M., UEYAMA, Y. & TAMAOKI, N. (1991). Electron microscopic identification of the intracellular secretion pathway of human G-CSF in a human tumor cell line: a comparative study with a Chinese hamster ovary cell line (IA1-7) transfected with human G-CSF cDNA, *Exp. Hematol.*, **19**, 768-772.

ASANO, S., URABE, A., OKABE, T., SATO, N., KONDO, Y., UEYUMA, Y., CHIBA, S., OHSAWA, N. & KOSAKA, K. (1977). Demonstration of granulopoietic factor(s) in the plasma of nude mice transplanted with a human lung cancer and in the tumor tissue. *Blood*, **49**, 845-852.

AVALOS, B.A., GASSON, J.C., HEDVAT, C., QUAN, S.G., BALDWIN, G.C., WEISBART, R.H., WILLIAMS, R.E., GOLDE, W.G. & DIPERSIO, J.F. (1990). Human granulocyte colony-stimulating factor: biologic activities and receptor characterization on hematopoietic cells and small cell lung cancer cell line. *Blood*, **75**, 851-857.

BERDEL, W.E., DANHAUSTER-RIEDL, S., STEILHAUSER, G. & WINTON, E.F. (1989). Various human hematopoietic growth factor (Interleukin-3, GM-CSF, G-CSF) stimulated clonal growth of nonhaematopoietic tumor cells. *Blood*, **73**, 80-83.

DEMETRI, G.D., BEATRICE, W.Z., RHEINWALD, J.D. & GRIFFIN, J.D. (1989). Expression of colony-stimulating factor genes by normal human mesothelial cells and human malignant mesothelioma cell lines *in vitro*. *Blood*, **74**, 940-946.

FIBBE, W.E., DAHA, M.R., HIEMSTRA, P.S., DUINKERKEN, N., LURVINK, E., RALPH, P., ALTROCK, B.W., KAUSHANSKY, K., WILLEMZE, R. FALKENBURG, J.H.F. (1989). Interleukin 1 and poly(rI), poly(rC) induce production of granulocyte CSF, macrophage CSF, and granulocyte-macrophage CSF by human endothelial cells. *Exp. Hematol.*, **17**, 229-234.

LEE, M.Y., KAUSHANSKY, K., JUDKINS, S.A., LOTTSFELDT, J.L., WAHEED, A. & SHADDUCK, R.K. (1989). Mechanisms of tumor-induced neutrophilia: Constitutive production of colony-stimulating factors and their synergistic actions. *Blood*, **74**, 115-122.

MANIATIS, T., FRITSH, E.F. & SAMBROOK, J. (1989). Molecular Cloning: A Laboratory Manual. Second edition. Cold Spring Harbor Laboratory Press: New York.

MCNICE, I., ANDREWS, R. & STEWART, M. (1989). Action of interleukin-3, G-CSF, and GM-CSF on highly enriched human hematopoietic progenitor cells: Synergistic interaction of GM-CSF plus G-CSF, *Blood*, **74**, 110-114.

METCALF, D. (1984). The Hematopoietic Colony Stimulating Factors. Elsevier Science Publishers: Amsterdam-New York.

MOTOJIMA, H., KOBAYASHI, T., SHIMANE, M., KAMACHI, S. & FUKUSIMA, M. (1989). Quantitative enzyme immunoassay for human granulocyte colony-stimulating factor (G-CSF). *J. Immunol. Meth.*, **118**, 187-192.

NAKAMURA, K., TAKAHASHI, T., TSUYUOKA, R., UEDA, Y., SUZUKI, A., OKUNO, Y., IHARA, Y., SEKO, S., OKADA, T., KUMAGAI, N., OYAIZU, T. & NISHIMURA, T. (1991). Identification of colony-stimulating factor activity in patients with malignant tumors associated with excessive leukocytosis. *Jpn. J. Clin. Oncol.*, **21**, 395-399.

NAGATA, S., TSUCHIYA, M., ASANO, S., KAZIRO, Y., YAMAZAKI, T., YAMAMOTO, O., HIRATA, Y., KUBOTA, N., OHEDA, M., NOMURA, H. & ONO, M. (1986). Molecular cloning and expression of cDNA for human granulocyte colony-stimulating factor. *Nature*, **319**, 415-417.

NOMURA, H., IMAZEKI, I., OHEDA, M., KUBOTA, N., TAMURA, M., ONO, M., UEYAMA, Y. & ASANO, S. (1986). Purification and characterization of human granulocyte colony-stimulting factor (G-CSF). *EMBO J.*, **5**, 871-876.

RENNICK, D., YANG, G., GEMMELL, L. & LEE, F. (1987). Control of hemopoiesis by a bone marrow stromal cell clone: Lipopolysaccharide and interleukin 1-inducible production of colony-stimulating factor. *Blood*, **69**, 682-691.

SATO, N., ASANO, S., UEYAMA, Y., MORI, M., OKABE, T., KONDO, Y., OHSAWA, N. & KOSAKA, K. (1979). Granulocytosis and colony-stimulating activity (CSA) and produced by a human squamous cell carcinoma. *Cancer*, **43**, 605-610.

SHIMAMURA, K., FUJIMOTO, J., HATA, J., AKATSUKA, A., UEYAMA, Y., WATANABE, T. & TAMAOKI, N. (1990). Establishment of specific monoclonal antibodies against recombinant human granulocyte colony-stimulating factor (hG-CSF) and their application for immunoperoxidase staining of paraffin-embedded sections. *J. Histochem. Cytochem.*, **38**, 283-286.

SHIRAFUJI, N., ASANO, S., MATSUDA, S., WATARI, K., TAKAKU, F. & NAGATA, S. (1989). A new bioassay for human granulocyte colony stimulating factor (G-CSF) using murine myeloblastic NFS-60 cells as targets and estimation of its level in sera from normal healthy persons and patients with infections and hematological disorder. *Exp. Hematol.*, **17**, 116-119.